# Multi-contrast delayed enhancement imaging (MCDE): accuracy and reproducibility compared to conventional SSFP and delayed hyperenhancement imaging

**DOI:** 10.1186/1532-429X-11-S1-P192

**Published:** 2009-01-28

**Authors:** Kim A Connelly, Jay S Detsky, John J Graham, Gideon Paul, Ram Vijayaraghavan, Rhonda Walcarius, Graham A Wright, Alexander J Dick

**Affiliations:** Sunnybrook HSC, Toronto, ON Canada

**Keywords:** Wall Motion, Cardiac Magnetic Resonance, Cardiac Magnetic Resonance Imaging, Inversion Pulse, Delay Enhancement Imaging

## Introduction

Cardiac magnetic resonance (CMR) imaging is an important tool in the assessment of cardiac function and viability, with prognostic implications for patients with ischemic heart disease [1]. Multi-contrast delayed enhancement imaging (MCDE) allows myocardial viability and wall motion to be assessed simultaneously by producing cardiac-phase-resolved images at multiple inversion times [2].

## Purpose

This study compared MCDE imaging to the conventional wall motion and viability CMR imaging for the evaluation of ejection fraction (EF), LV mass, LV end-diastolic volume (EDV) and infarct mass.

## Methods

**Figure 1:**
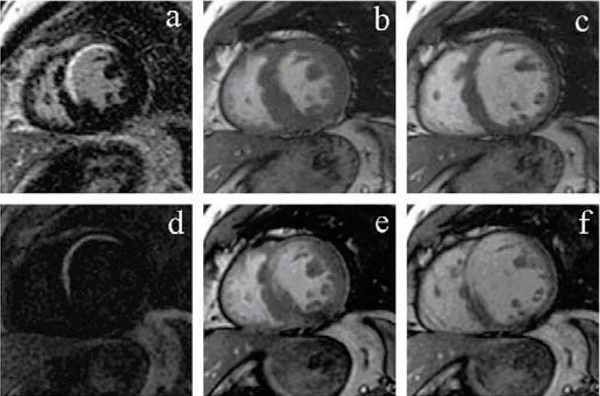
**A comparison between (a) IR-GRE delayed enhancement image, (b) conventional SSFP end-systolic wall motion image, (c) conventional SSFP end-diastolic wall motion image with (d) MCDE infarct-enhanced image, (e) MCDE end-systolic image, and (f) MCDE end-diastolic image**. Note that (d-f) are three of the 20 MCDE images acquired during a single breath-hold.

Forty-one patients with suspected myocardial infarction were studied. All patients underwent assessment of cardiac function (cine SSFP), followed by viability imaging ten minutes after administration of 0.2 mmol/kg Gd-DTPA using the inversion recovery gradient echo (IR-GRE) and MCDE sequences. The MCDE sequence uses a segmented SSFP acquisition following an inversion pulse; one inversion pulse is played out per heartbeat, and twenty images each at a different effective inversion time and cardiac phase are reconstructed. The position of the inversion pulse was placed just prior to diastole to produce infarct-enhanced images in diastole and systolic images with normal SSFP contrast in systole. MCDE therefore produces wall motion images in systole and viability images in diastole using a single breath-hold (see Figure 1).

## Results

CMR imaging was well tolerated in all subjects. Images from two patients were excluded from the final analysis as an incorrect placement of the inversion pulse for the MCDE sequence prevented systolic frame identification. Myocardial infarct was detected on delayed enhancement images in 24 patients, with associated wall motion abnormalities and a reduced EF. MCDE, IR-GRE, and SSFP imaging demonstrated excellent correlation for EF, LV infarct size, LV mass and LV EDV (all r>0.9, p < 0.001). Bland-Altman analysis demonstrated excellent agreement in the assessment of EF (bias = -2% (95% CI: -8% to 4%)), LV infarct size (bias = 0.2 g (-1.5 g to 2.0 g)) and LV mass (bias = 0.2 g (-18 g to 18 g)). Agreement was clinically acceptable for LV EDV (bias = -7 mL (-30 mL to 16 mL)). Inter and intra-observer variability was low between SSFP / IR-GRE and MCDE imaging.

## Conclusion

MCDE demonstrated excellent clinical agreement with conventional SSFP and IR-GRE imaging in the assessment of cardiac function and viability. MCDE provides wall motion and viability information during a single breath-hold with inherent spatial registration, as opposed to the cine SSFP and IR-GRE acquisitions which require separate breath-holds. MCDE imaging may be considered an alternative to conventional imaging.
